# Enhanced Tribocatalytic Degradation of Organic Pollutants by ZnO Nanoparticles of High Crystallinity

**DOI:** 10.3390/nano13010046

**Published:** 2022-12-22

**Authors:** Hua Lei, Xiaodong Cui, Xuchao Jia, Jianquan Qi, Zhu Wang, Wanping Chen

**Affiliations:** 1 School of Physics and Technology, Wuhan University, Wuhan 430072, China; 2 School of Natural Resources and Materials Science, Northeast University at Qinhuangdao, Qinhuangdao 066004, China; 3 Hubei Key Laboratory of Radiation Chemistry and Functional Materials, School of Nuclear Technology and Chemistry and Biology, Hubei University of Science and Technology, Xianning 437100, China

**Keywords:** tribocatalysis, friction energy harvesting, zinc oxide, dye decomposition

## Abstract

More and more metal oxide nanomaterials are being synthesized and investigated for degradation of organic pollutants through harvesting friction energy, yet the strategy to optimize their performance for this application has not been carefully explored up to date. In this work, three commercially available ZnO powders are selected and compared for tribocatalytic degradation of organic dyes, among which ZnO-1 and ZnO-2 are agglomerates of spherical nanoparticles around 20 nm, and ZnO-3 are particles of high crystallinity with a regular prismatic shape and smooth surfaces, ranging from 50 to 150 nm. Compared with ZnO-1 and ZnO-2, ZnO-3 exhibits a much higher tribocatalytic degradation performance, and a high degradation rate constant of 6.566 × 10^−2^ min^−1^ is achieved for RhB, which is superior compared with previous tribocatalytic reports. The stability and universality of ZnO-3 were demonstrated through cycling tests and degradation of different types of dyes. Furthermore, the mechanism of tribocatalysis revealed that h+ was the main active species in the degradation process by ZnO. This work highlights the great significance of high crystallinity rather than a large specific surface area for the development of high-performance tribocatalysts and demonstrates the great potential of tribocatalysis for water remediation.

## 1. Introduction

Environmental pollution and destruction, as well as the shortage of energy, are the most serious issues we are facing on a global scale [1,2,3,4]. For example, the release of textile waste in water resources poses a serious threat to human health and aquatic life [5,6,7]. Traditional adsorption, biological, and chemical methods still have limitations and shortcomings for dye degradation [8,9,10,11,12,13]. Therefore, technologies which are environmentally friendly, have low energy consumption, and are powered by renewable energy are highly desirable to eliminate dye pollutants.

Mechanochemical destruction based on ball milling, as one of the promising technologies for utilizing mechanical energy, has been widely studied for destructing chemicals in the wastes [14,15,16,17,18,19,20]. Rowlands et al. first reported the degradation of persistent organic pollutants by ball milling. It was found that all organic chlorides decomposed into monomeric carbon and inorganic chlorides after mixing DDT with CaO [21]. Zhou et al. realized the mechanochemical decomposition of methyl red by using a planetary mill with oxides of alkaline earth metals, such as CaO and MgO [22]. Results showed that the degradation rate of methyl red reached 91% after 6 h milling. However, the study of organic pollutant degradation is still limited to solid and non-aqueous phases, which means that the organic pollutants need to be enriched by adsorption first, thus making the ball milling method steps cumbersome [23,24,25]. Therefore, an alternative solution by exploiting tribocatalysis was proposed, which can directly degrade dyes in the liquid phase using friction energy. In 2019, Li et al. first reported that Ba_0.75_Sr_0.25_TiO_3_ (BST) nanoparticles successfully decomposed organic dye in the liquid phase by utilizing friction energy without enriching by adsorption [26]. BST nanoparticles in the PTFE-glass interface absorb the energy from PTFE friction against glass. Then, electron-hole pairs are excited, which, in turn, induce the redox chemical reactions for dyes decomposition. Subsequently, tribocatalytic technology has attracted special attention. A series of semiconductor materials, e.g., BiOIO_3_ [27], CdS [28], Bi_2_WO_6_ [29], BaTiO_3_ [30], Ba_4_Nd_2_Fe_2_Nb_8_O_30_ [31], TiO_2_ [32], Bi_12_TiO_20_ [33], NiCo_2_O_4_ [34], and Ba_2.5_Sr_2.5_Nb_8_Ta_2_O_30_ [35], were reported, thus demonstrating a great potential of tribocatalysis for dye decomposition.

It should be pointed out that tribocatalysis is quite similar to photocatalysis in utilizing clean energies from ambient environments. It is well known that a large specific surface area is of vital importance for photocatalysts. Similarly, a large specific surface area has generally been believed to be of vital importance for those materials employed in tribocatalysis, and only nanomaterials of large specific surface areas have been investigated for tribocatalysis up to date. Little attention has been paid to establishing a material optimization strategy specifically for tribocatalysis.

With well-known stability and safety, ZnO nanomaterials have demonstrated a great potential in environmental remediation through photocatalysis and piezocatalysis [36,37,38,39,40]. In the past couple of years, ZnO nanomaterials have also received much attention for environmental remediation through tribocatalysis [41,42,43]. In these investigations, all ZnO nanomaterials were carefully synthesized to achieve a large specific surface area. Though Fe-doped ZnO and biochar-ZnO composites were reported much more effective than their pure ZnO counterparts separately for tribocatalytic environmental remediation, it has to be pointed out that those pure ZnO nanomaterials actually had not been carefully optimized for tribocatalysis [42,43]. In this work, we have tried to explore the potential of pure ZnO nanomaterials in tribocatalytic environmental remediation by studying three kinds of quite different commercial ZnO powders: Two of which are agglomerates of 20 nm nanoparticles, and one is prismatic particles of high crystallinity with smooth surfaces, ranging from 50 to 150 nm. Results show that the tribocatalytic performance of ZnO nanomaterials has a strong correlation with their crystallinity rather than with their specific surface area. More than 99% of RhB was degraded within 90 min by the commercial ZnO powder of high crystallinity. The dye degradation is directly correlated with the friction between the ZnO nanoparticles and PTFE/glass interface, which was verified by using one PTFE-sealed bar with a degradation efficiency of 20%. This work highlights the critical importance of crystallinity rather than the specific surface area in material optimization for applications in tribocatalysis. More systematic investigations are highly desirable to establish a comprehensive material optimization strategy for tribocatalysis.

## 2. Materials and Methods

Rhodamine B (RhB, C_28_H_31_ClN_2_O_3_, Macklin, 99%), methyl orange (MO, C_14_H_14_N_3_NaO_3_S, Macklin, 99%), methyl violet (MV, C_24_H_27_N_3_·HCl, Macklin, 99%), methyl blue (MB, C_16_H_18_ClN_3_S·3H_2_O Macklin, 99%), terephthalic acid (TA, C_8_H_4_O_6_, Shanghai Aladdin Biochemical Technology Co., Ltd., Shanghai, China), ethylenediaminetetraacetic acid disodium salt dihydrate (C_10_H_14_N_2_Na_2_O_8_, Aladdin, 99%), tert-butanol (TBA, C_4_H_10_O, Sinopharm Chemical Reagent Co., Ltd., Shanghai, China 98.0%), silver nitrate (AgNO_3_, 99.8%, Sinopharm Chemical Reagent Co., Ltd.), p-Benzoquinone (BQ, C_6_H_4_O_2_, Macklin, 99%), three commercial zinc oxide powders (ZnO-1: Macklin, ZnO-2: Shanghai Xiao Huang Nano Technology CO., Ltd., Shanghai, China ZnO-3: Shanghai Nai Ou Nano Technology CO., Ltd., Shanghai, China). All reagents were of analytical grade and used without further purification. Deionized water was obtained from a Millipore Milli-Q purification system.

The tribocatalytic ability of these ZnO powders was measured through degradation of organic dye pollutants under magnetic stirring. In a typical experiment, 300 mg ZnO was dispersed in 30 mL 5 mg L^−1^ RhB aqueous solution in a flat-bottomed glass beaker (ϕ50 × 60 mm). The suspension was magnetically stirred through a homemade PTFE magnetic rotating disk at a speed of 400 rpm in dark at room temperature (25 °C). The details of the fabrication of the modified magnetic rotating disk have been described in our previous work [32]. During the tribocatalytic process, 2 mL solution was taken out every 15 min, followed by centrifugal separation to obtain the supernatant. The concentration of RhB was measured by recording the absorption spectra using a Shimadzu 2550 UV-Vis spectrometer on a range of 200–800 nm.

The active specie ·OH radical in dye degradation was identified by the reaction with terephthalic acid (TA) to produce 2-hydroxyterephthalic acid (TAOH) that has a characteristic photoluminescence (PL) signal centered at 425 nm by the excitation with the wavelength of 315 nm [44]. To further explore the tribocatalytic mechanism of ZnO nanoparticles, a trapping experiment using scavengers was performed. EDTA-2Na (6 mM), AgNO_3_ (6 mM), BQ (0.5 mM), and TBA (6 mM) were selected as scavengers for *h*^+^, *e*^−^, ·O_2_^−^, and ·OH, respectively. The reaction conditions were the same as the tribocatalysis reaction, except that additional radical scavengers were added to the reaction suspension.

## 3. Results

**Figure 1:**
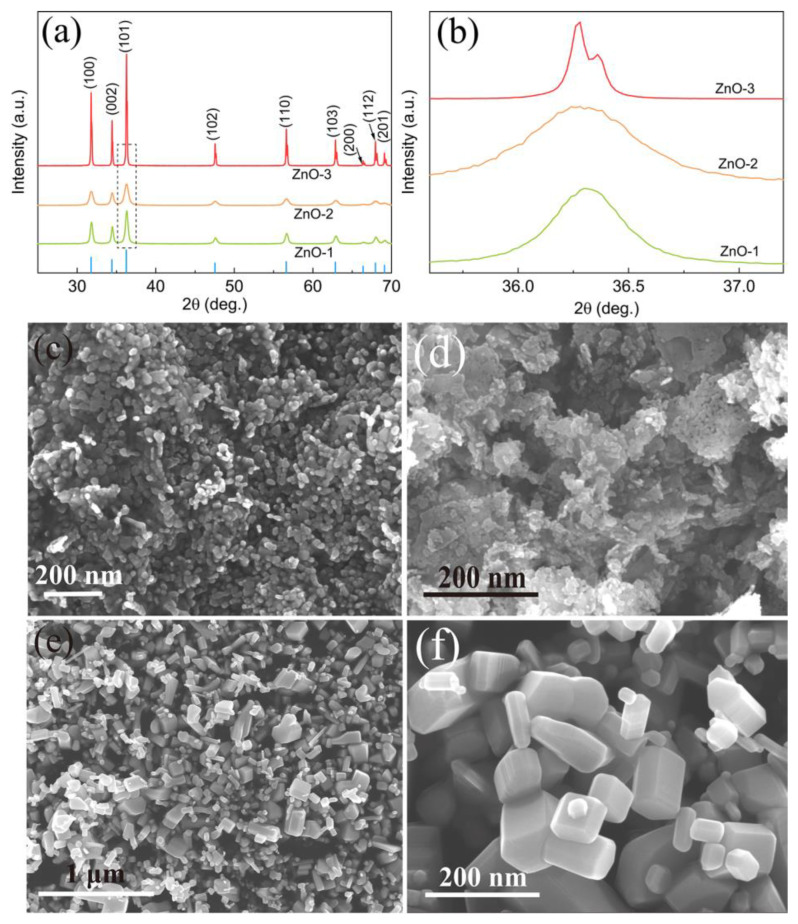
(**a**) XRD pattern of these ZnO powders; (**b**) normalized XRD result in the range of 35.5°–37.3°. SEM images of three kinds of ZnO powders: (**c**) ZnO-1; (**d**) ZnO-2; (**e**,**f**) ZnO-3.

X-ray Diffraction (XRD, SmartLab SE) technique was employed to explore the crystal structure of the three commercial ZnO powders. As shown in Figure 1a, all the diffraction peaks are seen at 31.8° (100), 34.4° (002), 47.5° (102), 56.6° (110), 62.9° (103), 66.4° (200), 67.9° (112), and 69.1° (201), which are matched well with the standard diffraction pattern of ZnO (JCPDS #36-1451) [45]. No obvious impurity peaks were detected, indicating these commercial ZnO samples have an acceptable purity. Meanwhile, the intensity of characteristic diffraction peaks of ZnO-3 is significantly increased compared to that of ZnO-1 and ZnO-2. Figure 1b shows the normalized XRD patterns in the range of 35.5°–37.3°, where it can be clearly seen that ZnO-1 and ZnO-2 (101) diffraction peak became much wider compared with ZnO-3, indicating a poorer crystallinity of ZnO-1 and ZnO-2.

The morphologies of these ZnO powders were investigated by Scanning Electron Microscopy (SEM, Zeiss GeminiSEM 500). As shown in Figure 1c,d, both ZnO-1 and ZnO-2 are agglomerates formed by numerous spherical nanoparticles with an average size of about 20 nm, where ZnO-2 presents an irregular flake shape (Figure 1d). For ZnO-3, the particles exhibit a regular prismatic bulk with smooth surfaces, diameter ranging from 50 nm to 150 nm, and are independently dispersed without any adhesion (Figure 1e,f), indicating a better crystallinity of ZnO-3, which is consistent with XRD results.

**Figure 2:**
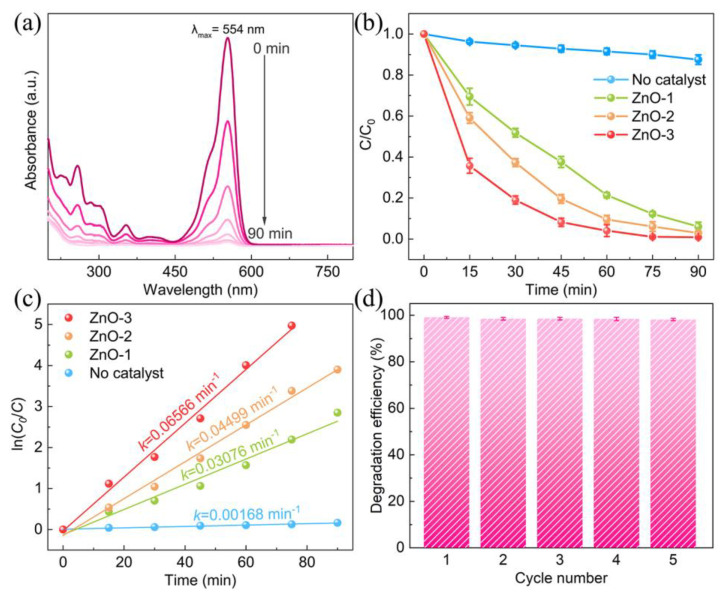
The catalytic properties of ZnO nanoparticles. (**a**) UV-Vis adsorption spectra of RhB (5 mg L^−1^) solution mediated by ZnO-3 under magnetic stirring with homemade PTFE disk for 90 min; (**b**) degradation efficiencies of RhB under magnetic by using a series of ZnO nanoparticles; (**c**) kinetic curves; (**d**) cycling stability.

The degradation experiments were performed using these three kinds of ZnO powders. Figure 2a presents the UV-Vis spectra of RhB solution by ZnO-3. With the prolongation of stirring time, the characteristic absorption peak at 554 nm decreased significantly and is nearly invisible after 60 min stirring. Meanwhile, the absorption in UV region declined, and no new absorption appeared, indicating that the aromatic ring of RhB was opened and decomposed into small molecules [46]. ZnO-1 and ZnO-2 also exhibit similar degradation curves, but RhB was not decolorized completely after 90 min. Figure 2b displays the (C/C_0_) vs. stirring time plots, where C and C_0_ are the in-progress and initial concentrations of the RhB solution. After 90 min stirring, only ~15% RhB degradation was obtained with no catalyst. With the addition of ZnO powders, the degradation efficiency is significantly improved. ZnO-3 exhibit a stronger tribocatalytic activity than that of ZnO-1 and ZnO-2. Subsequently, the reaction rate was evaluated through fitting data using the pseudo-first-order kinetics model ln(C_0_/C) = *k*t. As shown in Figure 2c, the good linearity of the fitted curves implies that the RhB degradation process obeys the pseudo-first-order reaction kinetics. The *k* value of ZnO-3 displayed the highest value (6.566 × 10^−2^ min^−1^), which is 1.5 and 2.1 times higher than that of ZnO-1 and ZnO-2, respectively, and also higher than that of all ZnO nanomaterials newly reported in the literature [41,42,43]. Thus, the result supports that the ZnO with high crystallinity possessed outstanding tribocatalytic activity for degrading dye pollutants.

A comparison with other tribocatalysts reported in the literature (see Table S1) proves that the ZnO powder of high crystallinity in this study is also highly outstanding among them for degrading RhB through harvesting friction energy. From the viewpoint of photocatalysis, the particles of ZnO-3 are too large in size to effectively absorb light and cannot exhibit an attractive photocatalytic performance. It can thus be clearly concluded that a quite different material optimization strategy should be developed for tribocatalysis from that for photocatalysis, which has been quite neglected up to date.

**Figure 3:**
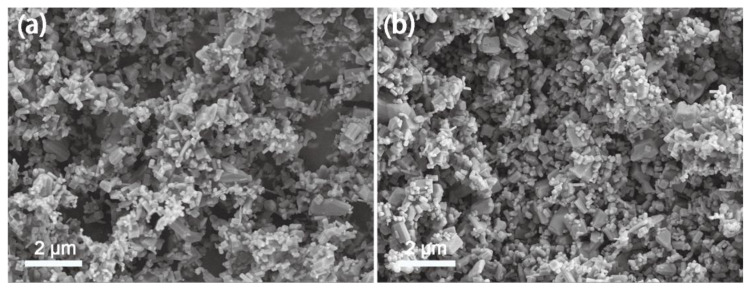
The SEM images of ZnO-3 powder: (**a**) As-received, and (**b**) after being magnetically stirred for five cycles.

The reusability and stability of the ZnO-3 were also investigated by repeated cycling tests with RhB solution. For each consecutive cycle, the recovered catalyst was reused with a fresh dye solution under identical experimental conditions. Figure 2d shows that the catalyst exhibited undiminished catalytic activity after five cycles. Meanwhile, the characteristic peak position did not change, and no new peak was found in XRD patterns of ZnO-3 before and after five cyclic tests (Figure S1). Moreover, SEM image shows that the morphology of the ZnO is almost unchanged after being magnetically stirred for five cycles (Figure 3), indicating that the structure of ZnO was highly stable in tribocatalytic process. As a matter of fact, materials of high crystallinity are usually more stable than those of poor crystallinity. Such high stability of ZnO-3 must be related with its high crystallinity. In other words, a high crystallinity is important for materials employed in tribocatalysis to achieve both high performance and high stability, which should receive critical attention in future material optimization strategies for tribocatalysis.

**Figure 4:**
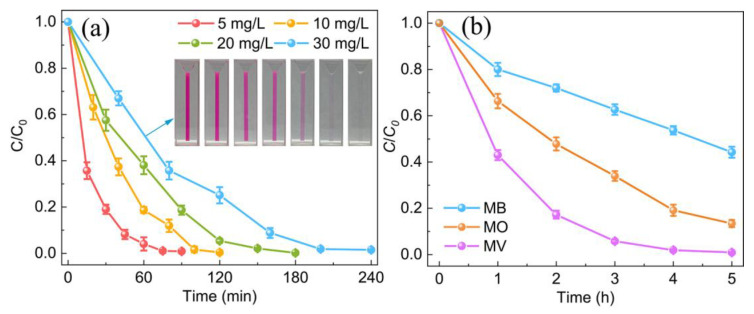
(**a**) Tribocatalytic degradation efficiency of RhB with different concentrations (5–30 mg L^−1^) by ZnO-3 (Inset: The optical photos of the RhB solution (30 mg L^−1^) from 0 to 240 min); (**b**) catalytic efficiencies of degradation of MB (10 mg L^−1^), MO (10 mg L^−1^), and MV (10 mg L^−1^), separately.

Previous reports of tribocatalysis for dye degradation have mostly focused on low concentrations of RhB (about 5 mg L^−1^), tribocatalytic performance for high initial concentration has been rarely reported. Therefore, a higher concentration of RhB and other different types of dye solution was selected to demonstrate the universality of ZnO-3 for tribocatalysis. As shown in Figure 4a, the degradation efficiency varies with the initial concentration of RhB solution. The initial concentration varies from 5 to 30 mg L^−1^, the time required for complete degradation increases gradually from 90 to 240 min. The illustration in Figure 3a shows the color change of 30 mg L^−1^ of RhB solution, where the naked ey can see that the solution color is gradually faded to colorless with the stirring time. Figure 4b exhibits catalytic performance of three different dyes, including MB (10 mg L^−1^), MO (10 mg L^−1^), and MV (10 mg L^−1^). After 5 h of stirring, the tribocatalytic degradation efficiencies of tribocatalysis MB, MO, and MV were approximately 55%, 85%, and 99%, respectively. These results demonstrate that tribocatalysis technology possesses good universality for dye degradation, suggesting the application potential of ZnO tribocatalysis in practice. As a matter of fact, tribocatalysis has even been reported capable of conversion of H_2_O and CO_2_ into flammable gases [47].

**Figure 5:**
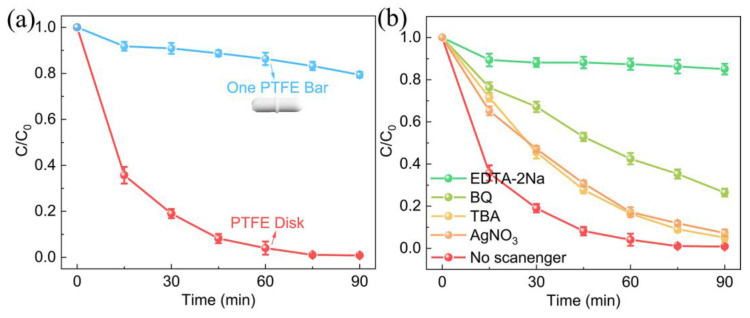
Comparison for tribocatalytic performance of ZnO-3: (**a**) With different shapes of PTFE-sealed magnetic rotating disk, (**b**) with the addition of various scavengers.

As we know, the key to tribocatalysis is the friction force between the catalyst and PTFE/glass interface. Therefore, a PTFE-sealed magnetic bar (ϕ5 × 20 mm) with flutes was used to avoid contact with the beaker. In comparison to the experiment using a homemade PTFE magnetic rotating disk, we can clearly find that the degradation efficiency of RhB using one PTFE-sealed magnetic bar was drastically inhibited, only 23% of RhB was degraded within 90 min, which reveals the activity of ZnO-3 depends significantly on the contact of magnetic PTFE disk and beaker (Figure 5a). Meanwhile, this result also indicated that the removal of RhB did not contribute to the adsorption effect on the catalyst.

**Figure 6:**
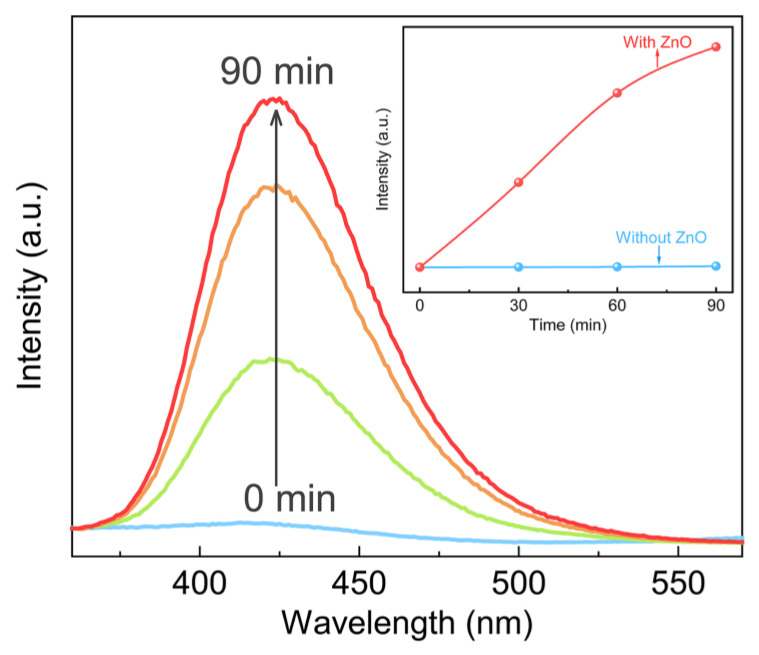
Fluorescence spectrum of 2-hydroxytereohthalic acid for ·OH detection under stirring for 90 min (Inset: The max intensity of 2-hydroxytereohthalic acid at 425 nm).

To better understand the mechanism of tribocatalysis, the trapping experiment of active species was performed to explore the effect of active species on the tribocatalysis process. As shown in Figure 5b, the tribocatalytic activity moderately decreased with the addition of BQ (·O_2_^−^ scavenger), TBA (·OH scavenger), and AgNO_3_ (*e*^−^ scavenger). After 90 min stirring, still 68%, 75%, and 97% of RhB was degraded, respectively. Whereas the tribocatalytic activity was remarkably inhibited after the addition of EDTA-2Na (*h*^+^ scavenger), only 15% RhB was degraded. The sequence for different ROS impact follows the order: EDTA-2Na (*h*^+^) > BQ(·O_2_^−^) > TBA(·OH) ~ AgNO_3_(*e*^−^). These results imply that *h*^+^ is the major active species, followed by ·O_2_^−^, ·OH, and *e*^−^. Figure 6 illuminates the dependence of fluorescence intensity of 2-hydroxytereohthalic acid on stirring time, which is ascribed to the accumulation of fluorescent 2-hydroxy TA formed by the interaction between ·OH and TA molecule [48]. With the increase of stirring time, the fluorescence intensity dramatically increases, suggesting the formation of ·OH radicals in the time of stirring.

Based on the above findings, the mechanism behind the tribocatalytic dye degradation by ZnO nanoparticles is proposed, and the whole process is described as the following Equations (1)–(4):

ZnO nanoparticles + friction → ZnO nanoparticles (*e*^−^ + *h*^+^) (1)

*e*^−^ + O_2_ → ·O_2_^−^ (2)

*h*^+^ + OH^−^ → ·OH (3)

*h*^+^ (main), *e*^−^/·O_2_^−^/·OH + dyes → degradation (4)

During dynamic friction, both phonons and electron-hole excitations can be excited in the surrounding media through the mechanical kinetic energy of the moving parts [49]. Under the stirring, ZnO absorbs the friction energy to excite the electron-hole pairs and further reacts with O_2_/OH^−^ to generate ·O_2_^−^/·OH [50]. Subsequently, the intermediate radicals react with dye molecules, resulting in degradation.

## 4. Conclusions

In summary, three commercial ZnO powders have been selected to explore tribocatalytic performance among different ZnO nanomaterials. ZnO nanoparticles with high crystallinity and large size exhibited a much higher tribocatalytic dye degradation efficiency than ZnO agglomerates. The RhB degradation efficiency for ZnO nanoparticles of high crystallinity reached 99% within 90 min under stirring with a rate constant of 6.566 × 10^−2^ min^−1^, which is superior compared with previous tribocatalysis reports. The ZnO nanoparticles exhibit good recycling utilization properties and high structural stability. The mechanism of tribocatalytic for dye degradation is revealed to be that *h^+^*, ·O_2_^−^, and ·OH radicals are generated during the stirring and account for the dye degradation. This investigation clearly demonstrates that an excellent tribocatalytic performance can be found in metal oxides of high crystallinity with relatively small specific surface area. A different material optimization strategy should be developed for tribocatalysis from that for tribocatalysis, in which much attention should be paid to material crystallinity.

## Data Availability

Not applicable.

## Supplementary Materials

The following supporting information can be downloaded at: https://www.mdpi.com/article/10.3390/nano13010046/s1, Figure S1: The XRD spectra of ZnO materials before and after five cyclic tests; Table S1: Comparison of the tribocatalytic performance of the reported photocatalysts for degrading dyes.
