# Supplementary material for: Enhanced Tribocatalytic Degradation of Organic Pollutants by ZnO Nanoparticles of High Crystallinity
Source: Nanomaterials (Basel). 2022 Dec 22;13(1):46. doi: 10.3390/nano13010046 (PMC9824812; doi:10.3390/nano13010046)
Supplement: Supplementary file 1 [file nanomaterials-13-00046-s001.zip › nanomaterials-2099145-supplementary.pdf]

nanomaterials
MDPI

Supplementary Materials

Enhanced tribocatalytic degradation of organic pollutants by ZnO nanoparticles of high crystallinity

Hua Lei [1], Xiaodong Cui[1], Xuchao Jia[1], Jianquan Qi [2], Zhu Wang[1,3], Wanping Chen[1,*]

[1] School of Physics and Technology, Wuhan University, Wuhan 430072, China
[2] School of Natural Resources and Materials Science, Northeast University at Qinhuangdao, Qinhuangdao 066004, Hebei Province, China
[3] Hubei Key Laboratory of Radiation Chemistry and Functional Materials, School of Nuclear Technology and Chemistry and Biology, Hubei University of Science and Technology, Xianning 437100, Hubei Province, China
[*] Correspondence: wpchen@whu.edu.cn

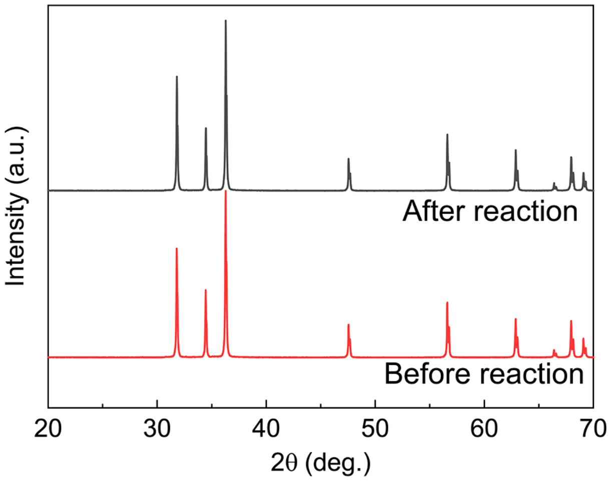

Figure S1. The XRD spetra of ZnO materials before and after five cyclic tests.

Table S1. Comparison of the tribocatalytic performance of the reported photocatalysts for degrading dyes .

Catalyst	Catalytic Conditions	Activities	Ref.
ZnO	PTFE Disk: Φ 40 mm; 400 rpm; RhB:5 mg L^{-1}, 30 mL	k = 0.066 min^{-1} 75 min 99.3%	This work
Biochar-ZnO	PTFE rod: Φ 8 × 45 mm; 450 rpm; RhB: 5 mg L^{-1}, 20 mL	k = 0.013 min^{-1} 75 min 90%	[1]
ZnO nanorods	1000 rpm; RhB: 5 mg L^{-1}, 20 mL	k = 0.082 h^{-1} 60 h 99.8%	[2]
Fe-ZnO	Ball milling; zirconia ball Φ 4 mm; RhB: 5 mg L^{-1}, 40 mL	k = 0.090 min^{-1} 12 min 75.8%	[3]
TiO_2	PTFE Disk: Φ 40 mm; 25 mg; RhB: 40 mg L^{-1}, 30 mL	80 min 73.2%	[4]
CdS	PTFE rod: Φ 8 × 25 mm; RhB: 5 ppm, 30 mL	7 h 98%	[5]
$BiOIO_3$	PTFE rod: Φ 8 × 35 mm; 500 rpm; RhB: 5 mg L^{-1}, 30 mL	k = 0.376 h^{-1} 12 h 99%	[6]
Bi_2WO_6	PTFE rod: Φ 8 × 35 mm; 500 rpm; RhB: 5 mg L^{-1}, 30 mL	k = 0.357 h^{-1} 12 h 99%	[7]
$NiCo_2O_4$	PTFE rod: Φ 6 × 20 mm; 400 rpm; RhB: 5 mg L^{-1}, 30 mL	k = 0.074 h^{-1} 56 h 98.6%	[8]

$Ba_4Nd_2Fe_2Nb_8O_{30}$	PTFE rod: Φ 9 × 25 mm; 300 rpm; RhB: 5 mg L^{-1}, 50 mL	120 min 97%	[9]
$BaSrTiO_3$	PTFE rod: Φ 8 × 25 mm; 300 rpm; RhB: 5 mg L^{-1}, 50 mL	180 min 99%	[10]

References

1. Hu, J.; Ma, W.; Pan, Y.; Chen, Z.; Zhang, Z.; Wan, C.; Sun, Y.; Qiu, C. Resolving the Tribo-catalytic reaction mechanism for biochar regulated Zinc Oxide and its application in protein transformation. *J. Colloid Interface Sci.* **2022**, 607, 1908–1918.
2. Zhao, J.; Chen, L.; Luo, W.; Li, H.; Wu, Z.; Xu, Z.; Zhang, Y.; Zhang., H.; Yuan, G.; Gao, J.; Jia, Y. Strong tribo-catalysis of zinc oxide nanorods via triboelectrically-harvesting friction energy. *Ceram. Int.* **2020**, 46, 25293–25298.
3. Hu, J.; Ma, W.; Pan, Y.; Cheng, Z.; Yu, S.; Gao, J.; Zhang, Z.; Wan, C.; Qiu, C. Insights on the mechanism of Fe doped ZnO for tightly-bound extracellular polymeric substances tribo-catalytic degradation: The role of hydration layers at the interface. *Chemosphere* **2021**, 276, 130170.
4. Cui, X.; Li, P.; Lei, H.; Tu, C.; Wang, D.; Wang, Z.; Chen, W. Greatly enhanced tribocatalytic degradation of organic pollutants by TiO_2 nanoparticles through efficiently harvesting mechanical energy. *Sep. Purif. Technol.* **2022**, 289, 120814.
5. Yang, B.; Chen, H.; Guo, X.; Wang, L.; Xu, T.; Bian, J.; Yang, Y.; Liu, Q.; Du, Y.; Lou, X. Enhanced tribocatalytic degradation using piezoelectric CdS nanowires for efficient water remediation. *J. Mater. Chem. C* **2020**, 8, 14845–14854.
6. Lei, H.; Wu, M.; Mo, F.; Ji, S.; Dong, X.; Wu, Z.; Gao, J.; Yang, Y.; Jia, Y., Tribo-catalytic degradation of organic pollutants through bismuth oxyiodate triboelectrically harvesting mechanical energy. *Nano Energy* **2020**, 78, 105290.
7. Wu, M.; Lei, H.; Chen, J.; Dong, X. Friction energy harvesting on bismuth tungstate catalyst for tribocatalytic degradation of organic pollutants. *J. Colloid. Interf. Sci.* **2021**, 587, 883–890.
8. Ruan, L.; Jia, Y.; Guan, J.; Xue, B.; Huang, S.; Wang, Z.; Fu, Y.; Wu, Z. Tribo-electro-catalytic dye degradation driven by mechanical friction using MOF-derived $NiCo_2O_4$ double-shelled nanocages. *J. Clean. Prod.* **2022**, 345, 131060.
9. Sun, C.; Guo, X.; Ji, R.; Hu, C.; Liu, L.; Fang, L.; Cheng, Z.; Luo, N. Strong tribocatalytic dye degradation by tungsten bronze $Ba_4Nd_2Fe_2Nb_8O_{30}$. *Ceram. Int.* **2021**, 47, 5038–5043.
10. Li, P.; Wu, J.; Wu, Z.; Jia, Y.; Ma, J.; Chen, W.; Zhang, L.; Yang, J.; Liu, Y. Strong tribocatalytic dye decomposition through utilizing triboelectric energy of barium strontium titanate nanoparticles. *Nano Energy* **2019**, 63, 103832.
